# Effects of Obesity on Pulmonary Inflammation and Remodeling in Experimental Moderate Acute Lung Injury

**DOI:** 10.3389/fimmu.2019.01215

**Published:** 2019-05-29

**Authors:** Lígia de A. Maia, Fernanda F. Cruz, Milena V. de Oliveira, Cynthia S. Samary, Marcos Vinicius de S. Fernandes, Stefano de A. A. Trivelin, Nazareth de N. Rocha, Marcelo Gama de Abreu, Paolo Pelosi, Pedro L. Silva, Patricia R. M. Rocco

**Affiliations:** ^1^Laboratory of Pulmonary Investigation, Carlos Chagas Filho Biophysics Institute, Federal University of Rio de Janeiro, Rio de Janeiro, Brazil; ^2^Department of Physiology and Pharmacology, Biomedical Institute, Fluminense Federal University, Niterói, Brazil; ^3^Pulmonary Engineering Group, Department of Anesthesiology and Intensive Care Therapy, University Hospital Dresden, Technische Universität Dresden, Dresden, Germany; ^4^Dipartimento di Scienze Chirurgiche e Diagnostiche Integrate, Università degli Studi di Genova, Genoa, Italy; ^5^IRCCS Ospedale Policlinico San Martino, Genoa, Italy

**Keywords:** obesity, neutrophil, macrophage, remodeling, acute respiratory distress syndrome, lung histology

## Abstract

Obese patients are at higher risk of developing acute respiratory distress syndrome (ARDS); however, their survival rates are also higher compared to those of similarly ill non-obese patients. We hypothesized that obesity would not only prevent lung inflammation, but also reduce remodeling in moderate endotoxin-induced acute lung injury (ALI). Obesity was induced by early postnatal overfeeding in Wistar rats in which the litter size was reduced to 3 pups/litter (Obese, *n* = 18); Control animals (*n* = 18) were obtained from unculled litters. On postnatal day 150, Control, and Obese animals randomly received *E. coli* lipopolysaccharide (ALI) or saline (SAL) intratracheally. After 24 h, echocardiography, lung function and morphometry, and biological markers in lung tissue were evaluated. Additionally, mediator expression in neutrophils and macrophages obtained from blood and bronchoalveolar lavage fluid (BALF) was analyzed. Compared to Control-SAL animals, Control-ALI rats showed no changes in echocardiographic parameters, increased lung elastance and resistance, higher monocyte phagocytic capacity, collagen fiber content, myeloperoxidase (MPO) activity, and levels of interleukin (IL-6), tumor necrosis factor (TNF)-α, transforming growth factor (TGF)-β, and type III (PCIII), and I (PCI) procollagen in lung tissue, as well as increased expressions of TNF-α and monocyte chemoattractant protein (MCP)-1 in blood and BALF neutrophils. Monocyte (blood) and macrophage (adipose tissue) phagocytic capacities were lower in Obese-ALI compared to Control-ALI animals, and Obese animals exhibited reduced neutrophil migration compared to Control. Obese-ALI animals, compared to Obese-SAL, exhibited increased interventricular septum thickness (*p* = 0.003) and posterior wall thickness (*p* = 0.003) and decreased pulmonary acceleration time to pulmonary ejection time ratio (*p* = 0.005); no changes in lung mechanics, IL-6, TNF-α, TGF-β, PCIII, and PCI in lung tissue; increased IL-10 levels in lung homogenate (*p* = 0.007); reduced MCP-1 expression in blood neutrophils (*p* = 0.009); decreased TNF-α expression in blood (*p* = 0.02) and BALF (*p* = 0.008) neutrophils; and increased IL-10 expression in monocytes (*p* = 0.004). In conclusion, after endotoxin challenge, obese rats showed less deterioration of lung function, secondary to anti-inflammatory and anti-fibrotic effects, as well as changes in neutrophil and monocyte/macrophage phenotype in blood and BALF compared to Control rats.

## Introduction

Approximately 20% of patients admitted to intensive care units (ICUs) are obese (1). Observational studies have shown an association between obesity and increased risk for acute respiratory distress syndrome (ARDS) (2). However, ARDS-related mortality is lower in obese compared to non-obese patients (2–5). In fact, all-cause ICU mortality is reduced in obese patients compared to similarly ill non-obese ones, a phenomenon that has been termed the “obesity paradox” or “reverse epidemiology” (5–7).

Currently, the pathophysiological mechanisms underlying the benefits of obesity in ARDS are poorly understood. It has been suggested that obesity is associated with continuous low-grade inflammation, which might protect the lungs from further insult, in the so-called “pre-conditioning cloud” (8). In experimental diet-induced obesity, neutrophil function including cytokine transcription, downstream signaling responses, and chemotaxis is impaired (9–11). Furthermore, in animals with acute lung injury (ALI), obesity has been shown to reduce the number of circulating monocytes and adhesion receptors, whereas alveolar macrophages develop a predominantly anti-inflammatory phenotype (12).

To our knowledge, however, no study has investigated whether obesity affects lung remodeling in experimental ALI. In this line, interstitial and intra-alveolar fibrosis are important determinants of ARDS outcome (13, 14).

We hypothesized that obesity would not only prevent lung inflammation, but also reduce remodeling after endotoxin challenge. To test this hypothesis, we assessed echocardiographic parameters, arterial blood gases, lung mechanics, and histology, as well as biological markers associated with inflammation and remodeling in lung tissue in obese and non-obese (Control) rats with and without ALI. Moreover, we evaluated gene expression of pro-inflammatory and anti-inflammatory mediators in neutrophils and macrophages/monocytes from BALF and blood.

## Materials and Methods

### Ethics Statement

This study was approved by the Ethics Committee of the Carlos Chagas Filho Biophysics Institute (CEUA-117/16), Federal University of Rio de Janeiro, Rio de Janeiro, Brazil. All animals received humane care in compliance with the “Principles of Laboratory Animal Care” formulated by the National Society for Medical Research and the *Guide for the Care and Use of Laboratory Animals* prepared by the National Academy of Sciences, USA. The present study followed the ARRIVE guidelines for reporting of animal research (15).

### Animal Preparation and Experimental Protocol

Wistar rats were kept in a temperature-controlled room (23–24°C) with artificial dark–light cycles (lights on at 7 a.m. and off at 7 p.m.). Virgin female rats, 3 months old, were caged with male rats in a 3:1 ratio. After mating, each female was placed in an individual cage with free access to water and food until delivery. To prevent any influence of litter size, we used only those dams whose litter size was 10–12 pups.

To induce early overfeeding (EO) during lactation, 3 days after birth, litters were culled to three males each (Obese) (16, 17). Ten-pup litters were used as the Control group. Two male rats were randomly chosen from each of the 18 different litters (18 obese animals and 18 control animals) for subsequent analysis. After postnatal day 21 (PN21), i.e., after the weaning period, both groups were given free access to water and a standard diet. From PN21 to PN180, offspring body weight (g) and food intake (g) were monitored every 7 days.

### Oral Glucose Tolerance Test

An oral glucose tolerance test (OGTT) was performed at PN150. After a 12-h fasting period, 50% glucose was administered in sterile saline (0.9% NaCl) through an oral gavage tube at 2 g/kg body weight. Blood was drawn from the tail tip of each animal and the plasma glucose concentration assessed with a commercial glucometer and matching glucose-oxidase reagent strips (Accu-Chek Advantage; Roche Diagnostics, Mannheim, Germany). Blood samples were collected before glucose was administered and 15, 30, 60, and 120 min after gavage (18).

### Acute Lung Injury Induction

On PN150, 200 μg *Escherichia coli* lipopolysaccharide (O55:B5, LPS Ultrapure, InvivoGen, Toulouse, France) (19, 20) suspended in saline solution to a total volume of 200 μl or an equivalent volume of saline (SAL) was instilled intratracheally in both Control and Obese animals, resulting in four experimental groups (*n* = 9, each): (1) Control-SAL; (2) Control-**ALI**; (3) Obese-SAL; and (4) Obese-**ALI**. After 24 h, echocardiography and lung mechanics were analyzed, and biological samples were collected. Lungs were stored for further evaluation of histology, enzyme-linked immunosorbent assay (ELISA), and myeloperoxidase (MPO) activity. Gene expression was also quantified in macrophages and neutrophils obtained from blood and BALF.

### Echocardiography

For assessment of cardiac function, animals were anesthetized by intraperitoneal injection of ketamine 75 mg/kg and midazolam 2 mg/kg. The ventral thorax was shaved and animals were placed in dorsal recumbency. An expert blinded to group allocation (N.N.R.) performed transthoracic echocardiography using a system (UGEO HM70A, Samsung, São Paulo, Brazil) equipped with a linear phased-array probe (8–13 MHz). Images were obtained from the parasternal long-axis (PLAX) and short-axis views. The following parameters were obtained from PLAX (M-mode): left ventricle diameter, interventricular septum thickness, and posterior wall thickness. Isovolumic relaxation time was defined the interval between the end of aortic ejection and the onset of mitral inflow. Pulsed-wave Doppler was used to measure pulmonary acceleration time (PAT) to pulmonary ejection time (PET), the ratio of which (PAT/PET, an indirect index of pulmonary arterial hypertension) was then calculated (21, 22). All parameters followed recent recommendations for cardiovascular measurements (23, 24).

### Lung Mechanics

After echocardiography, animals were paralyzed by intravenous administration of pancuronium bromide (2 mg/kg, Cristália, Itapira, SP, Brazil), tracheotomized, and mechanically ventilated (Servo-I, MAQUET, Sweden) in volume-controlled mode with a tidal volume (V_T_) 7 ml/kg, a respiratory rate adjusted to a minute ventilation of 150 ml/min, fraction of inspired oxygen (FiO_2_) 0.4, and positive end-expiratory pressure (PEEP) 5 cmH_2_O for 5 min.

A pneumotachograph (internal diameter = 1.5 mm, length = 4.2 cm, distance between side ports = 2.1 cm) was connected to the tracheal cannula for airflow measurements. The pressure gradient across the pneumotachograph was determined using a SCIREQ differential pressure transducer (UT-PDP-02, SCIREQ, Montreal, Canada). V_T_ was calculated by digital integration of the flow signal (25). Airway pressure was measured with a SCIREQ differential pressure transducer (UT-PDP-75, SCIREQ, Montreal, Canada). A 30 cm-long water-filled catheter (PE-205, Becton, Dickinson and Company) with side holes at the tip, connected to a differential pressure transducer (UT-PL-400, SCIREQ, Montreal, Quebec, Canada), was used to measure the esophageal pressure, as described elsewhere (26). Airflow, airway, and esophageal pressures were continuously recorded throughout the experiments with a computer running custom-made software written in LabVIEW (National Instruments, Austin, TX). Transpulmonary pressure was calculated by the difference between airway and esophageal pressures. All signals were filtered, amplified (SC-24, SCIREQ, Montreal, QC, Canada), and sampled at 200 Hz with a 12-bit analog-to-digital converter (National Instruments; Austin, TX, USA). Lung elastance (E,_L_) and resistance (R,_L_) were computed offline using a routine written in MATLAB (Version R2007a; The Mathworks Inc., Natick, Massachusetts, USA).

After assessment of lung mechanics, heparin (1,000 IU) was injected into the tail vein, and animals were killed by injection of sodium thiopental (60 mg/kg). Visceral fat (mesenteric, epididymal, and retroperitoneal white adipose tissue) was immediately excised and weighed for evaluation of central adiposity.

### Lung Histology

The trachea was clamped at end-expiration (PEEP = 5 cmH_2_O), and the lungs were removed *en bloc*. Quantitative evaluation of lung structure was performed according to American Thoracic Society/European Respiratory Society guidelines (27). The right lower lung was frozen in liquid nitrogen. Frozen lungs were fixed in Carnoy's solution (ethanol/chloroform/acetic acid at a 70:20:10 ratio) at −70°C for 24 h. Solutions with progressively increasing concentrations of ethanol at −20°C were then substituted for Carnoy's solution until a 100% ethanol concentration was reached. The tissue was maintained at −20°C for 4 h, warmed to 4°C for 12 h, and then allowed to reach and remain at room temperature for 2 h (13, 28, 29). After fixation, the tissue was embedded in paraffin. Slices 4 μm thick were obtained by means of a microtome and stained with hematoxylin-eosin.

Lung morphometric analysis was performed using an integrating eyepiece with a coherent system consisting of a grid with 100 points and 50 lines of known length coupled to a conventional light microscope (Olympus BX51; Olympus Latin America, Brazil). The volume fraction of the lung occupied by collapsed alveoli was determined by the point-counting technique at 200 × magnification across 10 random, non-coincident microscopic fields (27, 30). Briefly, points falling on collapsed pulmonary areas were counted and divided by the total number of points in each microscopic field. Fraction area of neutrophils in the alveolar septa were evaluated at 1,000 × magnification and determined by the point-counting technique. To quantify interstitial edema, five arteries were transversely sectioned. The number of points falling on areas of perivascular edema and the number of intercepts between the lines of the integrating eyepiece and the basal membrane of the vessels were counted. The interstitial perivascular edema index was calculated as follows: number of points^1/2^/number of intercepts (31).

In order to evaluate the remodeling process, the amount of collagen fibers (stained by the Picrosirius polarization method) was computed in alveolar septa. The images were generated by a microscope (Axioplan, Zeiss) connected to a digital camera (Sony Trinitron CCD, Sony, Tokyo, Japan) and fed into a computer through a frame grabber (Oculus TCX, Coreco, St. Laurent, QC, Canada) for offline processing. The threshold for collagen was established after enhancement of contrast up to the point where fibers were easily identified as birefringent bands at 400 × magnification, using ImagePro Plus 7.1 Software (Media Cybernetics, Silver Spring, MD, USA) (32–34). The areas occupied by collagen fibers were measured by digital densitometric recognition, divided by the tissue area of each zone of interest, and expressed as the percentage of collagen fiber in the alveolar septal area. Bronchi and blood vessels were carefully avoided during the measurements. Over 20 fields were analyzed, thus reducing any possible bias when computing collagen fiber content due to alveolar collapse. Lung histology was analyzed by an investigator blinded to group assignment (M.V.F.).

### Enzyme-Linked Immunosorbent Assay (ELISA)

The right upper lung was immediately frozen in liquid nitrogen and stored at −80°C for ELISA. Interleukin (IL)-6, Tumor necrosis factor (TNF)-α, IL-10, and transforming growth factor (TGF)-β levels were quantified by ELISA in the lung tissue homogenate. All procedures were done in accordance with the manufacturer's protocol (Peprotech, London, UK), normalized to total protein as assessed by Bradford's reagent (Sigma-Aldrich, St. Louis, MO, USA), and expressed as pg/mg. ELISA analysis was done by an investigator blinded to group assignment (L.A.M.).

### Myeloperoxidase Activity

Myeloperoxidase (MPO) was extracted from homogenized right middle lung samples by suspending the material in 0.5% hexadecyltrimethylammonium bromide (Sigma Chemical Co, St. Louis, MO) in 50 mmol/l potassium phosphate buffer, pH 6.0, before sonication in an ice bath for 10 s. The samples were frozen and thawed three times, after which sonication was repeated. Suspensions were then centrifuged at 15,000 g for 30 min and the resulting supernatant further assayed.

MPO activity was assayed spectrophotometrically. Briefly, 0.1 ml of supernatant was mixed with 2.9 ml of 50 mmol/l phosphate buffer, pH 6.0, containing 0.167 mg/ml *o*-dianisidine dihydrochloride (Sigma Chemical) and 0.0005% hydrogen peroxide (Sigma Chemical). The change in absorbance at 460 nm was measured using a spectrophotometer (V-Max; Molecular Devices) running SoftMax pro 4.0 software. MPO activity was then derived from the observed change in absorbance per minute, further normalized to total protein as assessed by Bradford's reagent (Sigma-Aldrich, St. Louis, MO, USA), an expressed as units of MPO activity per mg protein. This process was conducted by an investigator blinded to group assignment (L.A.M.).

### Molecular Biology Analysis of Lung Tissue

Quantitative real-time reverse transcription polymerase chain reaction (RT-PCR) was performed to measure type I (PCI) and III (PCIII) procollagen—biological markers associated with fibrogenesis—in the lungs. The primer sequences were: PCIII, sense 5′-ACC TGG ACC ACA AGG ACA C-3′, antisense 5′-TGG ACC CAT ACC TTT C-3′; PCI, (sense 5′-AGA AGT CTC AAG ATG GTG GCC G-3′ and antisense 5′-GGT CAC GAA CCA CGT TAG CAT C-3′). Central slices of right lung and diaphragm were cut, collected in cryotubes, flash-frozen by immersion in liquid nitrogen, and stored at −80°C. Total RNA was extracted from frozen tissues using the RNeasy Plus Mini Kit (Qiagen, Hilden, Germany) for the lungs and RNeasy Fibrous Tissue Mini Kit (Qiagen, Hilden, Germany) for the diaphragm, following the manufacturer's recommendations. The RNA concentration was measured by spectrophotometry in a Nanodrop ND-1000 system. First-strand cDNA was synthesized from total RNA using a Quantitec reverse transcription kit (Qiagen, Hilden, Germany). Relative mRNA levels were measured by SYBR green detection in an ABI 7,500 real-time PCR system (Applied Biosystems, Foster City, CA, USA). Samples were measured in triplicate. For each sample, the expression of each gene was normalized to that of the housekeeping gene *36B4* (acidic ribosomal phosphoprotein P0) and expressed as fold change relative to Control-SAL, using the 2^−ΔΔ*Ct*^ method, where ΔCt = Ct (reference gene)—Ct (target gene). All analyses were performed by one of the authors (F.F.C.), who was blinded to group assignment.

### *In vitro* Analysis of Gene Expression in Neutrophils and Macrophages

Cells were extracted from blood and BALF. Blood was collected in a heparinized tube by cardiac puncture and centrifuged at 5,000 g at 4°C for 5 min; plasma was retrieved, and blood cells used for monocyte and neutrophil isolation. BALF was collected by flushing the left lung three times with sterile PBS through the tracheal cannula. BALF was then centrifuged at 1,200 g for 5 min, supernatant was discarded, and cells in the pellet were resuspended and used for macrophage and neutrophil isolation. For isolation of neutrophils and macrophages, Percoll gradient was used, as previously described (35). Then, total RNA was extracted from neutrophils and macrophages with the ReliaPrep RNA Cell Miniprep System (Promega, Madison, WI, USA), per manufacturer recommendations. The total RNA concentration was measured by spectrophotometry in a Nanodrop ND-1000 system. First-strand cDNA was synthesized from total RNA using the High-Capacity cDNA Reverse Transcription Kit (Applied Biosystems, Foster City, CA). Relative mRNA levels were measured with Bryt™ Green dye (Promega, Fitchburg, WI) in a Mastercycler ep Realplex system (Eppendorf, Hamburg, Germany). All experiments were performed in triplicate. Monocyte chemoattractant protein-1 (MCP-1) and TNF-α gene expressions were measured in neutrophils, while IL-6 and IL-10 were evaluated in macrophages. The relative level of each gene was normalized to the housekeeping gene *36B4* (acidic ribosomal phosphoprotein P0) and expressed as fold change relative to the Control-SAL group, using the 2^−ΔΔ^Ct method, where ΔCt = Ct_(target gene)_–Ct_(housekeeping gene)_ (36). All *in vitro* analyses of gene expression were done by an investigator blinded to group assignment (F.F.C.).

### Neutrophil Migration Capability

The ability of neutrophils derived from blood or adipose tissue to migrate toward a chemotactic agent was monitored using the Cytosensor Cell Migration Assay (Molecular Devices, California, USA), a microplate assay which measures migration of cells from an upper chamber through a 3-μm pore membrane into a lower chamber containing or not LPS 1 nmol/l. Neutrophils (5 × 10^4^) were placed in the upper chamber and microplates incubated at 37°C, 5% CO_2_. After 1 h, cells were counted for comparison purposes, and the percent increase in the number of cells that migrated after exposure to the LPS stimulus was calculated (37).

### Macrophage Phagocytosis

Phagocytic activity of macrophages isolated from blood and adipose tissue was determined in a fluorescence reader using the pHrodo Red Zymosan A Bioparticles Phagocytosis kit (Life technologies, Oregon, USA). The engulfed bacteria fluoresce when in the low-pH environment of the acidified phagocytic compartment. Macrophages (10^4^ cells) were mixed with Zymosan bioparticles (1 mg/ml) and incubated for 2 h at 37°C. Samples without bioparticles constituted the negative control (37).

### Statistical Analysis

The sample size was calculated on the basis of pilot studies which detected differences in IL-6 between Obese-ALI and Control-ALI animals. A sample size of eight animals per group would provide the appropriate power (1–β = 0.8) to identify significant differences in IL-6 (adjusted α = 0.025 for two comparisons), taking into account an effect size *d* = 2.0, a two-sided *t-*test, and a sample size ratio of 1 (G^*^Power 3.1.9.2, University of Düsseldorf, Düsseldorf, Germany). Each variable was tested for normality using the Kolmogorov–Smirnov test. Data are presented as mean ± standard deviation (SD). Comparison between the Control vs. Obese groups at baseline (before ALI induction) was done using Student's *t*-test. After ALI induction, data were compared using two-way ANOVA followed by Bonferroni's correction. The two-stage linear step-up procedure of Benjamini et al. (38), which controls the false discovery rate of a family of hypothesis tests and protects against type II errors, was also done. All tests were performed in GraphPad Prism version 6.07 (GraphPad Software, La Jolla, CA, USA). The significance level was set at 5%.

## Results

### Obese-SAL vs. Control-SAL

**Figure 1:**
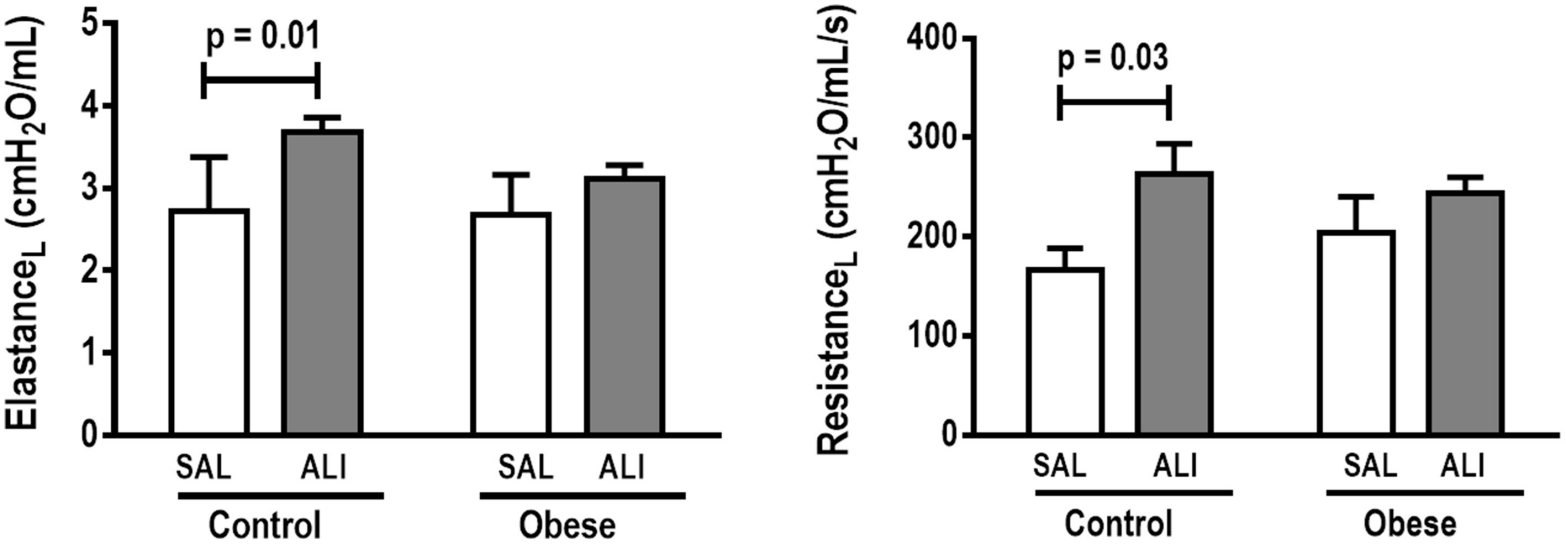
Lung mechanics. Dynamic elastance and resistance (measured with PEEP = 5 cmH_2_O) in Control and Obese animals treated with saline (SAL) or endotoxin (ALI) intratracheally. Values are means ± SD of nine animals/group.

At weaning (postnatal day 21 day), Obese-SAL rats had greater body weight compared to Control-SAL animals. In adulthood, body weight, mesenteric, epididymal, and retroperitoneal fat mass, and fasting hyperglycemia were greater in Obese-SAL than Control-SAL (Supplemental Figure S1). Oral glucose tolerance test results did not differ between groups. In Obese-SAL compared to Control-SAL animals, the internal diameter of the left ventricle was smaller, while interventricular septum thickness, posterior left ventricle wall thickness, and isovolumic relaxation time were greater; PAT/PET ratio did not differ (Supplemental Figure S2). The fraction area of alveolar collapse, interstitial edema, the percentage of neutrophils in alveolar septa (Table 1), and MPO activity (Figure 3) were worse in Obese-SAL than Control-SAL animals, but gas exchange (Supplemental Table S1), lung mechanics (Figure 1), and collagen fiber content (Table 1) did not differ significantly.

**Table 1:** Lung morphometry.

	**Control**			**Obese**		
	**SAL**	**ALI**	***p***	**SAL**	**ALI**	***p***
Collapse (%)	4.8 [2.9–5.5]	12.4 [9.7–19.2]	<0.0001	9.0 [6.1–10.5]^*^	18.8 [16.0–23.2]^#^	0.002
Interstitial edema	0.47 ± 0.25	0.94 ± 0.16	0.0002	0.89 ± 0.15^*^	0.80 ± 0.12	0.397
Neutrophils (%)	1.2 ± 0.6	5.7 ± 1.8	<0.0001	3.1 ± 0.6^*^	5.5 ± 1.6	0.001
Collagen fibers (%)	2.0 [1.7–2.9]	6.9 [5.8–7.5]	<0.0001	3.2 [2.1–4.1]	3.5 [3.1–3.9]^#^	0.43

*Fraction area of collapsed alveoli, interstitial edema, percentage of neutrophils and collagen fibers in Control and Obese animals subjected to intratracheal instillation of saline (SAL) or endotoxin (ALI). Values represent median (interquartile range) or mean (SD) of nine animals/group. Interstitial edema represented as a dimensionless quantity*.

* *vs. Control-SAL (p < 0.05)*;

# *vs. Control-ALI (p < 0.05)*.

**Figure 2:**
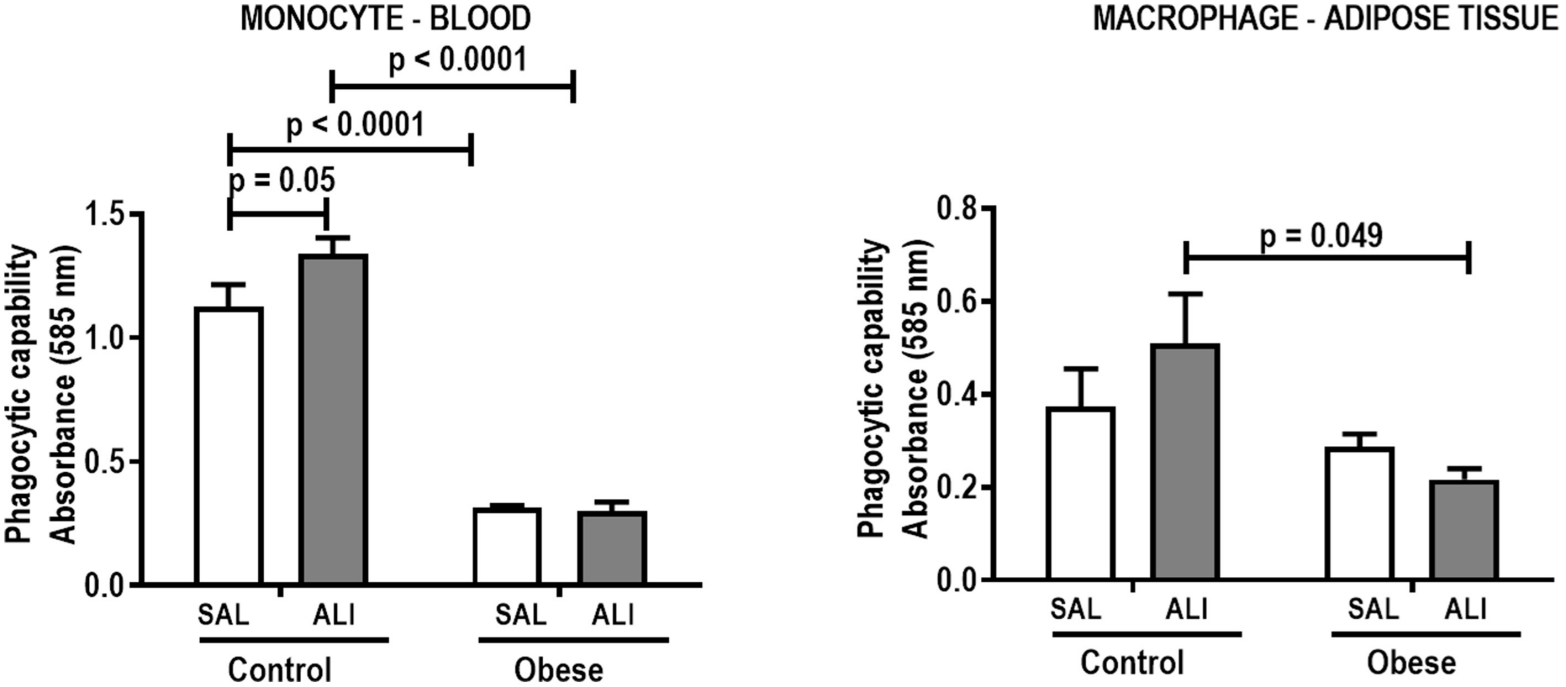
Phagocytic capability of monocytes (blood) and macrophages (adipose tissue) from Control and Obese animals treated with saline (SAL) or endotoxin (ALI) intratracheally. Values are means + SD of six animals/group.

**Figure 3:**
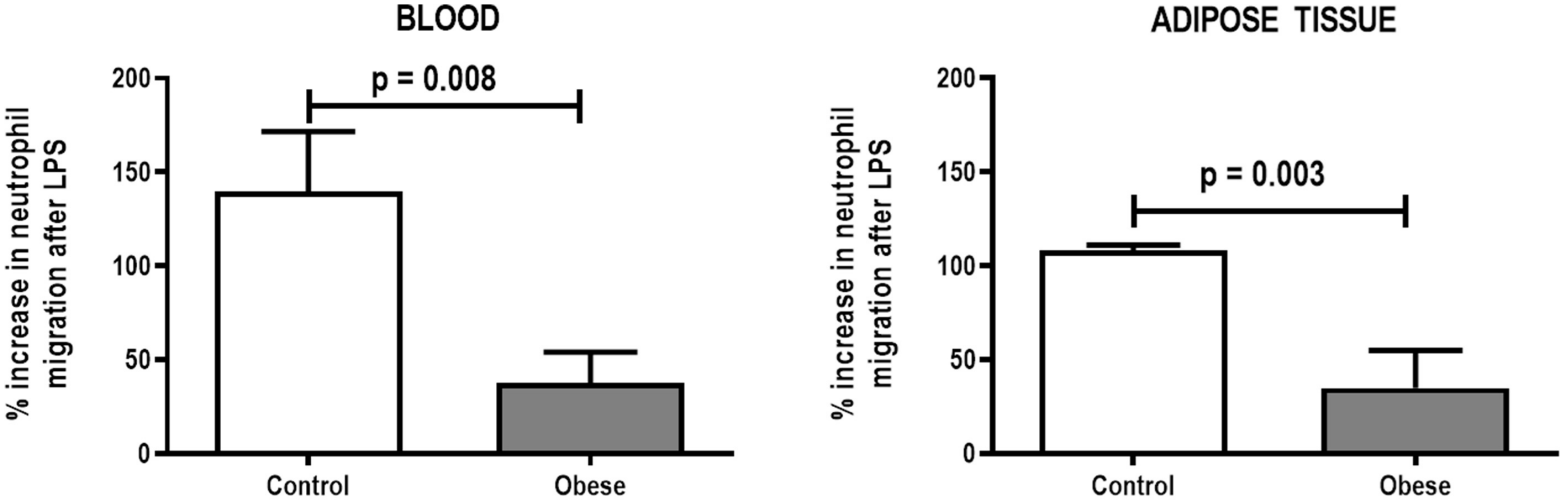
Percent increase in neutrophil migration from blood and adipose tissue of Control and Obese animals after endotoxin (LPS) exposure. Values are means + SD of six animals/group.

Monocyte phagocytic capability in the blood was reduced in Obese-SAL (Figure 2). Obese animals also exhibited reduced neutrophil migration compared to Controls (Figure 3).

**Figure 4:**
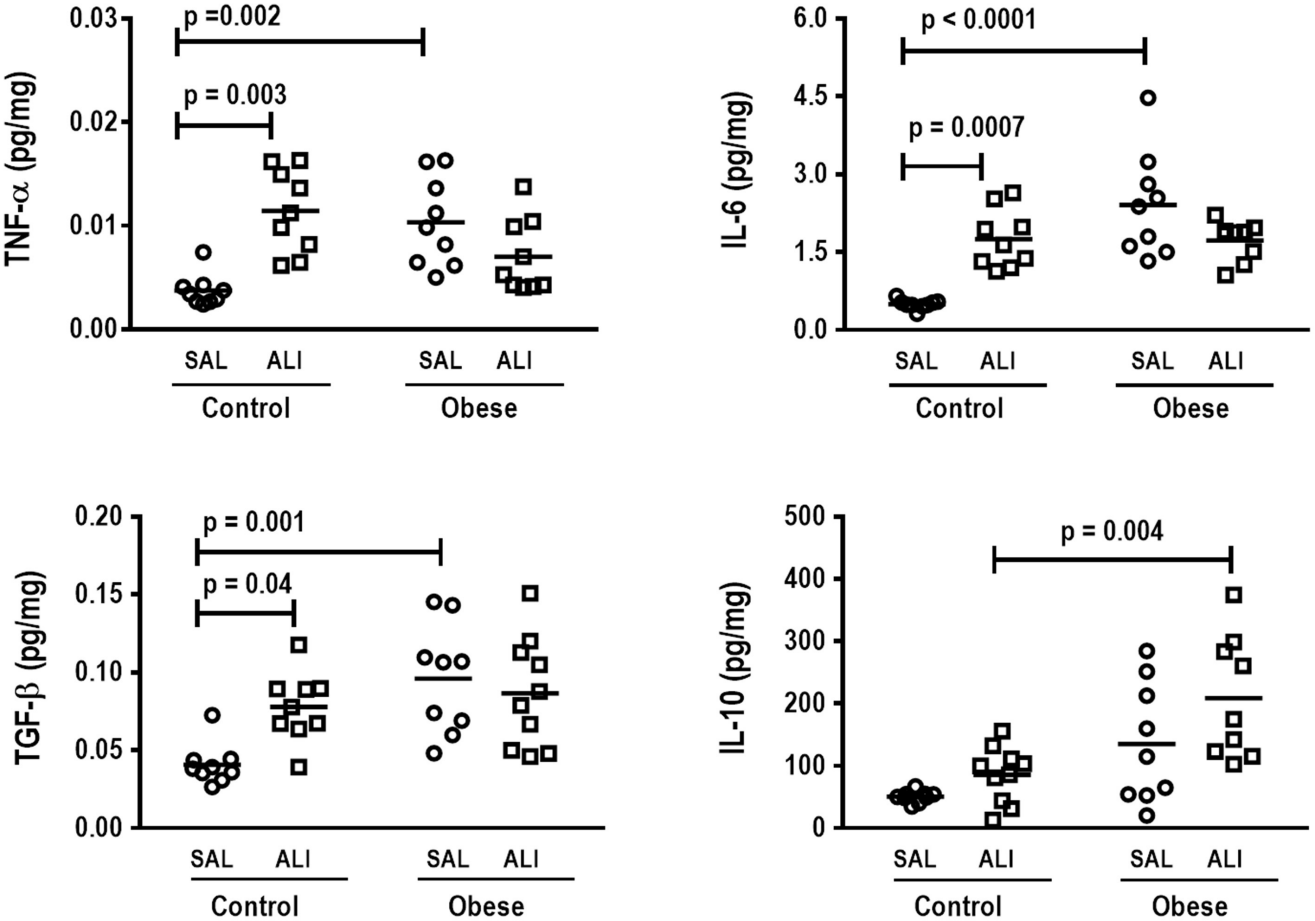
Protein levels of TNF-α, IL-6, TGF- β, and IL-10 in lung tissue. Levels of tumor necrosis factor (TNF)-α, interleukin (IL)-6, transforming growth factor (TGF)-β, and IL-10 in lung tissue from Control and Obese animals treated with saline (SAL) or endotoxin (ALI) intratracheally. Scatter dot plot indicates individual animals and median of nine animals/group.

**Figure 5:**
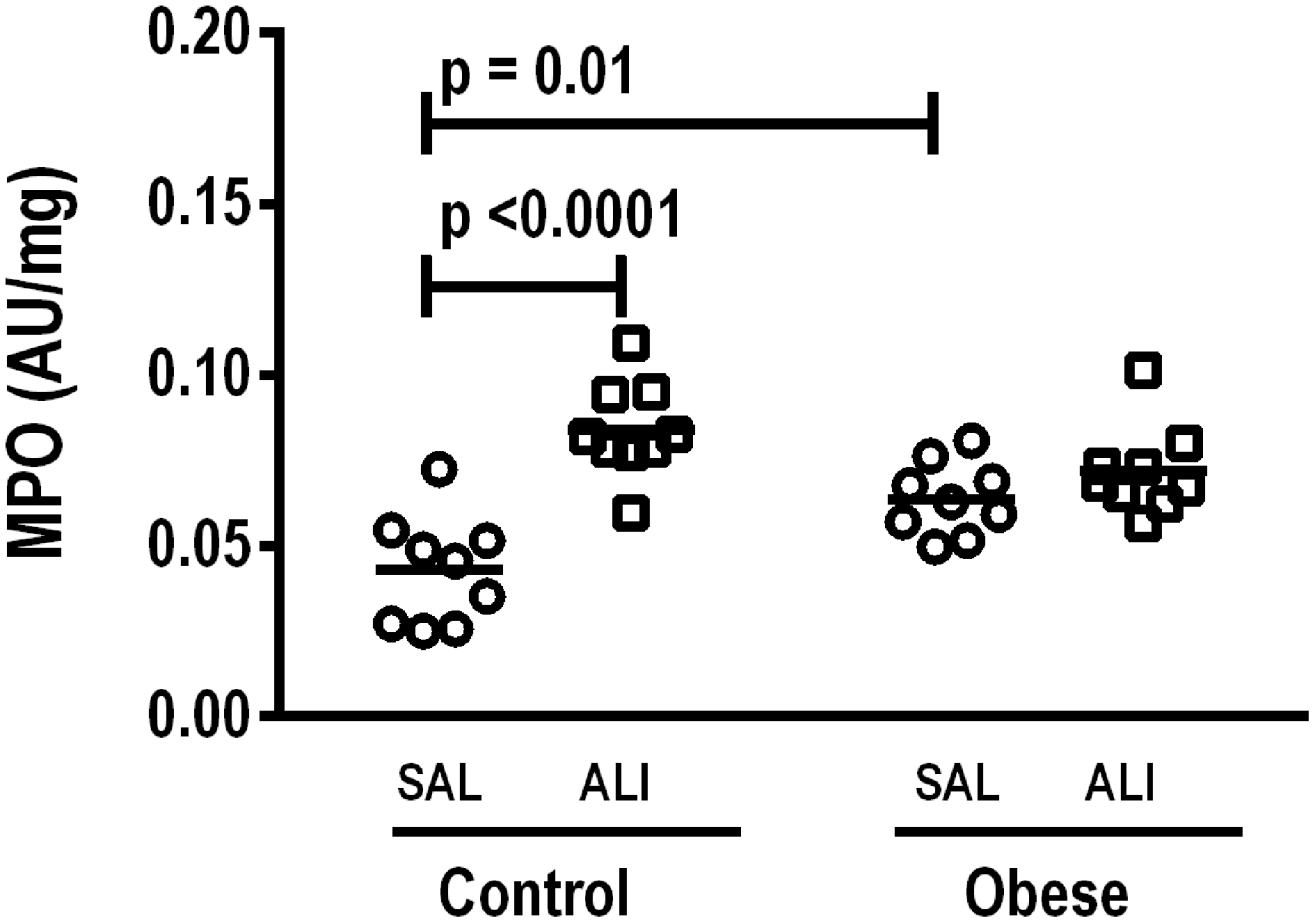
Myeloperoxidase (MPO) activity/mg total protein in lung homogenate. MPO analyzed in lung tissue computed from Control and Obese animals treated with saline (SAL) or endotoxin (ALI) intratracheally. Scatter dot plot indicates individual animals and median of nine animals/group.

**Figure 6:**
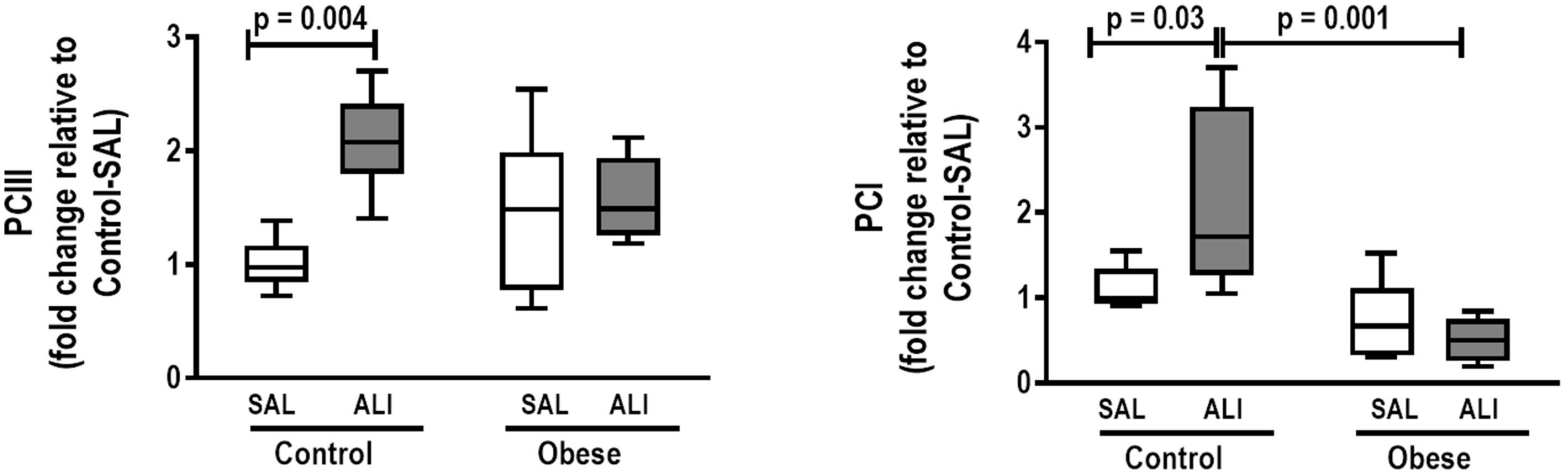
Real-time polymerase chain reaction analysis of type III (PCIII) and type I (PCI) procollagen from Control and Obese animals treated with saline (SAL) or endotoxin (ALI) intratracheally. Relative gene expression was calculated as the difference in average gene expression levels compared with the reference gene *36*β*4* (acidic ribosomal phosphoprotein P0) and expressed as fold change relative to Control-SAL. Boxes show the interquartile range (25–75%), whiskers denote the range (minimum–maximum), and horizontal lines represent the median in nine animals/group.

**Figure 7:**
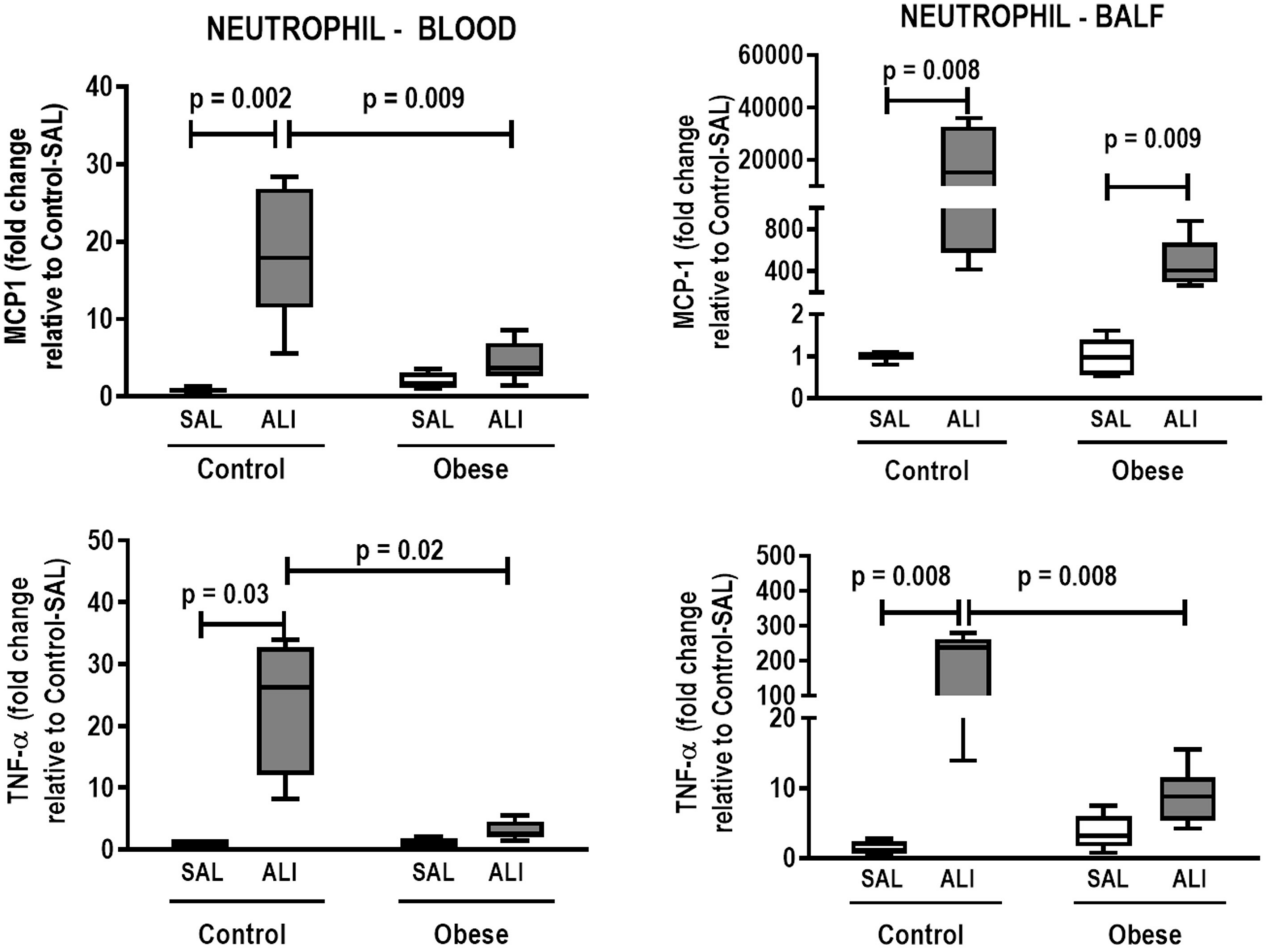
Expression of biological markers. Real-time polymerase chain reaction analysis of monocyte chemoattractant protein-1 (MCP-1) and tumor necrosis factor (TNF)-α from Control and Obese animals treated with saline (SAL) or endotoxin (ALI) intratracheally. Left panel: Blood neutrophils. Right panel: BALF neutrophils. Relative gene expression was calculated as the difference in average gene expression levels compared with the reference gene *36*β*4* (acidic ribosomal phosphoprotein P0) and expressed as fold change relative to Control-SAL. Boxes show the interquartile range (25–75%), whiskers denote the range (minimum–maximum), and horizontal lines represent the median in nine animals/group.

**Figure 8:**
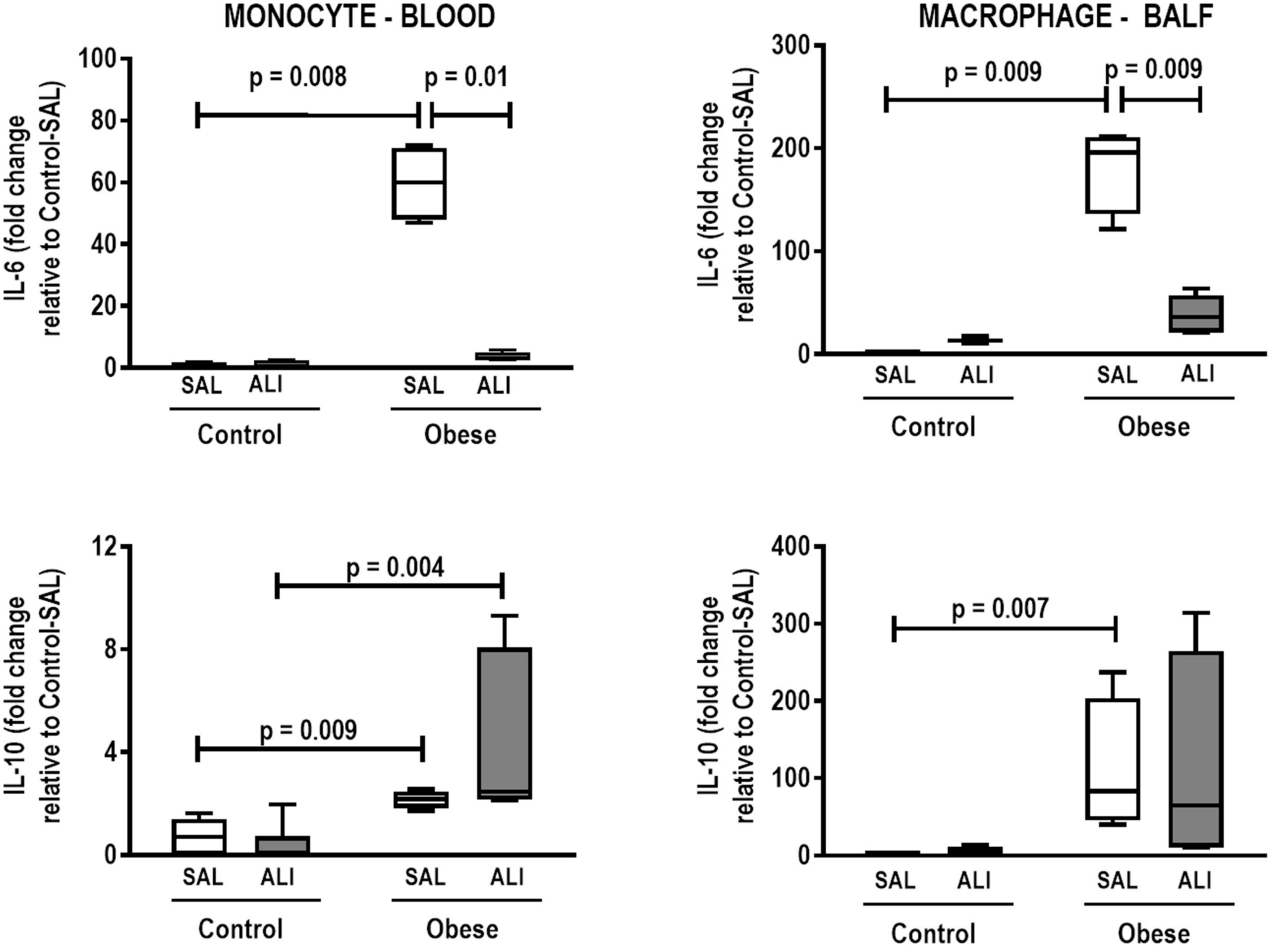
Expression of biological markers. Real-time polymerase chain reaction analysis of interleukin (IL)-6 and IL-10 from Control and Obese animals treated with saline (SAL) or endotoxin (ALI) intratracheally. Left panel: Blood monocytes. Right panel: BALF macrophages. Relative gene expression was calculated as the difference in average gene expression levels compared with the reference gene *36*β*4* (acidic ribosomal phosphoprotein P0) and expressed as fold change relative to Control-SAL. Boxes show the interquartile range (25–75%), whiskers denote the range (minimum–maximum), and horizontal lines represent the median in nine animals/group.

In lung tissue homogenates, Obese-SAL animals exhibited increased levels of TNF-α, IL-6, and TGF-β (Figure 4) as well as MPO activity (Figure 5) compared to their Control-SAL counterparts, while no significant changes were observed in levels of IL-10 (Figure 4), as well as gene expression of PCI and PCIII (Figure 6). In neutrophils from blood and BALF, expression of MCP-1 and TNF-α did not differ between Obese-SAL and Control-SAL animals (Figure 7). However, monocytes/macrophages obtained from blood/BALF showed increased expression of IL-6 and IL-10 in the Obese-SAL group (Figure 8).

### Obese-ALI vs. Control-ALI

Interventricular septum thickness and posterior wall thickness were increased in Obese-ALI as compared to Control-ALI animals, while PAT/PET ratio was reduced (Supplemental Figure S2). Alveolar collapse was greater and collagen fiber content was lower in Obese-ALI than Control-ALI animals (Table 1). Interstitial edema and the percentage of neutrophils did not differ between Obese-ALI and Control-ALI.

As in the SAL groups, monocyte (blood) and macrophage (adipose tissue) phagocytic capabilities were lower in Obese-ALI compared to Control-ALI animals (Figure 2), and Obese animals presented reduced neutrophil migration compared to Control (Figure 3).

Gas exchange (Supplemental Table S1), lung mechanics (Figure 1), levels of IL-6, TNF-α, and TGF-β in lung tissue homogenate (Figure 4), MPO activity (Figure 5), and PCIII mRNA expression (Figure 6) did not differ significantly between the two groups, whereas protein level of IL-10 was higher (Figure 5) and PCI mRNA expression was lower (Figure 6) in Obese-ALI than Control-ALI. In Obese-ALI animals, MCP-1 expression was reduced in blood neutrophils, whereas TNF-α expression was decreased in neutrophils from both blood and BALF (Figure 7). Monocytes expressed more IL-10 in Obese-ALI than Control-ALI animals (Figure 8).

### Control-ALI vs. Control-SAL and Obese-ALI vs. Obese-SAL

Oxygenation was reduced (Supplemental Table S1) whereas lung elastance and resistance decreased (Figure 1). The phagocytic capacity of monocytes was higher in Control-ALI than Control-SAL, but not in Obese-ALI than Obese-SAL (Figure 2). Interstitial edema, collagen fiber content (Table 1), levels of IL-6, TNF-α, and TGF-β in lung tissue homogenate (Figure 4), MPO activity in lung tissue (Figure 5), and gene expression of types I and III procollagen (Figure 6) were increased in Control-ALI compared to Control-SAL, but not in Obese-ALI compared to Obese-SAL.

The fraction area of alveolar collapse and the percentage of neutrophils were increased in Control-ALI compared to Control-SAL and Obese-ALI compared to Obese-SAL (Table 1), but the percent increase was greater in Control-ALI vs. Control-SAL (2.6-fold increase and 4.8-fold increase, respectively) than in Obese-ALI vs. Obese-SAL (2-fold increase and 1.8-fold increase, respectively).

TNF-α expression in blood and BALF neutrophils was increased in Control-ALI compared to Control-SAL, but not in Obese-ALI compared to Obese-SAL. MCP-1 gene expression was increased in the blood and BALF of Control-ALI compared to Control-SAL animals, while in Obese-ALI, MCP-1 gene expression was increased only in BALF neutrophils (Figure 7).

Even though IL-6 and IL-10 expression did not change in monocytes or macrophages from blood or BALF in Control-ALI compared to Control-SAL animals, IL-6 expression was reduced in both monocytes and macrophages from blood and BALF in the Obese-ALI group compared to Obese-SAL (Figure 8).

## Discussion

When challenged with intratracheal administration of endotoxin, obese animals responded differently from non-obese (Control) animals in several aspects: no impairment in oxygenation and lung mechanics; reduced phagocytic capacity of monocytes (in blood) and macrophages (from adipose tissue); reduced collagen fiber content; comparable interstitial edema, percentage of neutrophils in alveolar septa, TNF-α, IL-6, and TGF-β levels and MPO activity in lung tissue homogenates; higher IL-10 levels in lung tissue; decreased gene expression of PCI; reduced expression of MCP-1 in blood neutrophils and of TNF-α in both blood and BALF; and increased expression of IL-10 in monocytes. Moreover, obesity also led to decreased neutrophil migration.

Several strengths of the present study are worth noting. First, we used a metabolic programming model of obesity, which, compared to other models based on dietary interventions, better resembles the major hallmarks of clinical obesity in humans (16, 17, 39–41). In fact, dietary interventions may influence the inflammatory response, which would be highly undesirable in this field of research (42). Second, animals were ventilated for a short period and a protective ventilation strategy was used, minimizing potential influences of mechanical ventilation on the parameters of interest. Finally, our model of obesity was associated with increased posterior left ventricle mass and left ventricle wall thickness with a concomitant reduction of left ventricle internal diameter, which closely resemble the cardiovascular changes seen in human obesity (43). Even though alveolar collapse was greater in obese than control animals, because of increased pleural pressure associated with increased surface tension due to surfactant dysfunction and decreased lung volumes (44), lung mechanics, and gas exchange were unaffected, since a certain threshold of pulmonary damage must be crossed to modify lung function (45). Similarly, the increased percentage of neutrophils was not high enough to impair lung function.

Our model presented some of the main features of experimental endotoxin-induced ALI (46), such as: (1) histological evidence of tissue injury characterized by accumulation of neutrophils in the interstitial space, thickening of the alveolar wall, interstitial edema, and atelectasis; (2) an inflammatory response characterized by increased lung myeloperoxidase activity (a surrogate marker of neutrophil activity) and concentrations of proinflammatory cytokines in lung tissue; and (3) evidence of physiological dysfunction characterized by hypoxemia and changes in lung mechanics (25, 46). In our Control-ALI animals, oxygenation was reduced and lung elastance and resistance were greater than in their Control-SAL counterparts, likely due to alveolar collapse, interstitial edema, neutrophil infiltration, and collagen deposition (46). Moreover, increased levels of TNF-α and IL-6 as well as MPO activity in lung tissue, associated with increased gene expressions of PCI and PCIII in lung tissue, as well as MCP-1 and TNF-α in neutrophils from blood and BALF, were observed in the Control-ALI group (47).

In obese animals, the ALI induction protocol used in this study did not result in increased levels of TNF-α, IL-6, or TGF-β in lung tissue, nor did it affect MPO activity; this is consistent with previous studies (9, 10). Additionally, TNF-α and MCP-1 were no longer expressed in neutrophils from blood and BALF. This absence of a pro-inflammatory response in obese animals following endotoxin challenge may be associated with several factors. These include endotoxin-induced lipolysis leading to an increase in circulating fatty acids (48), which bind to the macrophage toll-like receptor 4 (TLR-4), thus limiting release of pro-inflammatory mediators, which reduces lung inflammation (49); impairment in the migration of neutrophils from bone marrow to target tissues (9); and the possibility that obese individuals have a metabolic reserve to counteract the increased catabolic stress of ARDS, because of additional energy stores in the form of adipose tissue (9). In fact, neutrophils from obese rats shown decreased chemotactic migration to LPS compared to control animals. It is possible that an obesity-related neutrophil impairment contributes to the unexpected lower inflammation observed in Obese-ALI (8, 9). Levels of the anti-inflammatory cytokine IL-10 in lung tissue, as well as gene expression of IL-10 in monocytes, were both increased in Obese-ALI to Control-ALI animals; this may be attributed to increased M2 macrophage activation due to the increased total number of adipocytes (50–52). Moreover, the phagocytic capabilities of monocytes and macrophages play important roles in the regulation of inflammatory response (53). In our study, we observed reduced phagocytic capacity in blood monocytes and adipose-tissue macrophages from Obese-ALI compared to Control-ALI animals. Mice with specific knockdown of TLR-4 in bronchial or alveolar epithelial cells are known to exhibit significantly attenuated airway inflammation, barrier disruption, and lung edema, as well as prolonged survival, in response to LPS exposure (54). It is possible that the TLR-4 inactivation by fatty acids released from LPS-induced lipolysis (48) may blunt the phagocytic capability of monocytes and macrophages in obese animals.

In addition to the impact of obesity on lung inflammation, remodeling was also affected; neither collagen fiber deposition nor expression of TGF-β, a marker of fibrogenesis (55), were increased in Obese-ALI animals. To our knowledge, this was the first study to evaluate the purported effects of obesity on lung remodeling in ALI. The absence of differences in lung mechanics between Obese-ALI and Obese-SAL may be associated with a balance between the increase in alveolar collapse and reduction in interstitial edema and collagen fiber content.

This study represents a step forward in understanding the mechanisms of “obesity paradox” in ARDS patients, even though it remains a controversial subject with several confounding factors (5–7).

## Limitations

This study presents some limitations. First, we used a postnatal early overnutrition model; thus, our results cannot be extrapolated to other situations, including genetic variations and diet-induced obesity. Second, even taking the shorter life span of rats into account, the duration of exposure of our animals to obesity-induced changes differed from the time course of obesity in humans. Accordingly, several comorbidities that are determined by the aging process could not be reproduced. Third, it must be kept in mind that moderate ALI was induced by intratracheal LPS instillation, and our results do not necessarily apply to other models of experimental ALI, different degrees of ALI severity, nor to clinical ARDS. Fourth, obesity may increase the size of the lungs, leading to different responses to endotoxin exposure; however, in our study, no significant difference in lung size was observed between Control and Obese animals on chest CT (See Supplemental Figure S3). Fifth, the absence of significant differences in some parameters between Control-ALI and Obese-ALI rats may be attributed to the fact that obese animals already had a baseline inflammatory state; thus, endotoxin challenge in these animals did not lead to any significant further increase in lung inflammation and remodeling, as would be expected. Sixth, damage to the alveolar–capillary membrane was evaluated by measuring the degree of interstitial edema and neutrophil infiltration in alveolar septa. Further studies are required to better understand the pathogenesis of alveolar–capillary membrane failure in obese and non-obese rats with and without ALI. Seventh, it has already been demonstrated that elevated leptin levels may impair pulmonary host defenses (56) and increase release of pro-inflammatory mediators (57, 58). This is another mechanism that should be evaluated in future research. Eighth, two-way ANOVA followed by Bonferroni's correction protects from type I error, while increasing vulnerability to type II errors. Thus, the two-stage linear step-up procedure of Benjamini et al. (38) was performed additionally to protect against type II errors. No significant differences were observed between the results obtained using the two tests. Finally, this is a preliminary study and more research should be carried out using higher doses of LPS or another model of ALI, different strategies of mechanical ventilation, and more specific biological readouts to better understand how obesity affects lung inflammation and remodeling in experimental ALI.

In conclusion, after endotoxin challenge, obese compared to non-obese (Control) rats exhibited less deterioration of lung function, associated with an anti-inflammatory effect and reduced lung fibrosis, as well as changes in neutrophil and monocyte/macrophage phenotype in blood and BALF. Therefore, obesity appears protective against experimental ALI.

## Ethics Statement

This study was approved by the Ethics Committee of the Carlos Chagas Filho Biophysics Institute (CEUA-117/16), Federal University of Rio de Janeiro, Rio de Janeiro, Brazil. All animals received humane care in compliance with the Principles of Laboratory Animal Care formulated by the National Society for Medical Research and the Guide for the Care and Use of Laboratory Animals prepared by the National Academy of Sciences, USA.

## Author Contributions

LM, PS, and PR: study concept and design, drafting the manuscript, and statistical analysis. LM, FC, CS, MO, MF, ST, and NR: acquisition of data. LM, MO, MF, ST, NR, PP, MG, PS, and PR: analysis and interpretation of data. LM, FC, PP, MG, PS, and PR: critical revision of the manuscript for important intellectual content. All authors contributed to manuscript revision, read and approved the submitted version.

### Conflict of Interest Statement

The authors declare that the research was conducted in the absence of any commercial or financial relationships that could be construed as a potential conflict of interest.
