# Supplementary material for: Effects of Obesity on Pulmonary Inflammation and Remodeling in Experimental Moderate Acute Lung Injury
Source: Front Immunol. 2019 May 29;10:1215. doi: 10.3389/fimmu.2019.01215 (PMC6593291; doi:10.3389/fimmu.2019.01215)
Supplement: Supplementary file 1 [file Table_1.DOCX]

**Supplemental Table S1:** Arterial blood gases

|  | **Control** | | **Obese** | |
| --- | --- | --- | --- | --- |
|  | **SAL** | **ARDS** | **SAL** | **ARDS** |
| **pHa** | 7.42 ± 0.07 | 7.44 ± 0.03 | 7.40 ± 0.04 | 7.43 ± 0.06 |
| **PaCO_2_ (mmHg)** | 37 ± 7 | 32 ± 4 | 35 ± 4 | 33 ± 4 |
| **PaO_2_/FiO_2_** | 470 ± 30 | 327 ± 34* | 390 ± 19 | 406 ± 18 |
| **HCO_3_ (mmol/L)** | 24 ± 1 | 23 ± 2 | 22 ± 2 | 23 ± 2 |

Arterial blood gases in Control and Obese animals subjected to intratracheal instillation of saline (SAL) or endotoxin (ARDS). Values are means ± standard deviation (SD) of 9 animals/group. pHa: arterial pH; PaCO_2_: partial pressure of carbon dioxide in arterial blood; PaO_2_/FiO_2_: partial pressure of oxygen in arterial blood divided by fraction of inspired oxygen; HCO_3_: bicarbonate. * *vs* Control-SAL (p < 0.05).
