# Supplementary material for: Effects of Obesity on Pulmonary Inflammation and Remodeling in Experimental Moderate Acute Lung Injury
Source: Front Immunol. 2019 May 29;10:1215. doi: 10.3389/fimmu.2019.01215 (PMC6593291; doi:10.3389/fimmu.2019.01215)
Supplement: Supplementary file 2 [file Data_Sheet_1.docx]

**Supplemental Figure S1:** Upper panel: Progression of body weight during lactation and after weaning until age 147 days. Middle panel: Fasting blood glucose, glucose tolerance curve, and area under the curve. Lower panel: Visceral fat mass of Control and Obese rats (n=18 each). Values are means; vertical bars represent the standard deviation.
