# Supplementary material for: Effects of Obesity on Pulmonary Inflammation and Remodeling in Experimental Moderate Acute Lung Injury
Source: Front Immunol. 2019 May 29;10:1215. doi: 10.3389/fimmu.2019.01215 (PMC6593291; doi:10.3389/fimmu.2019.01215)
Supplement: Supplementary file 3 [file Data_Sheet_2.docx]

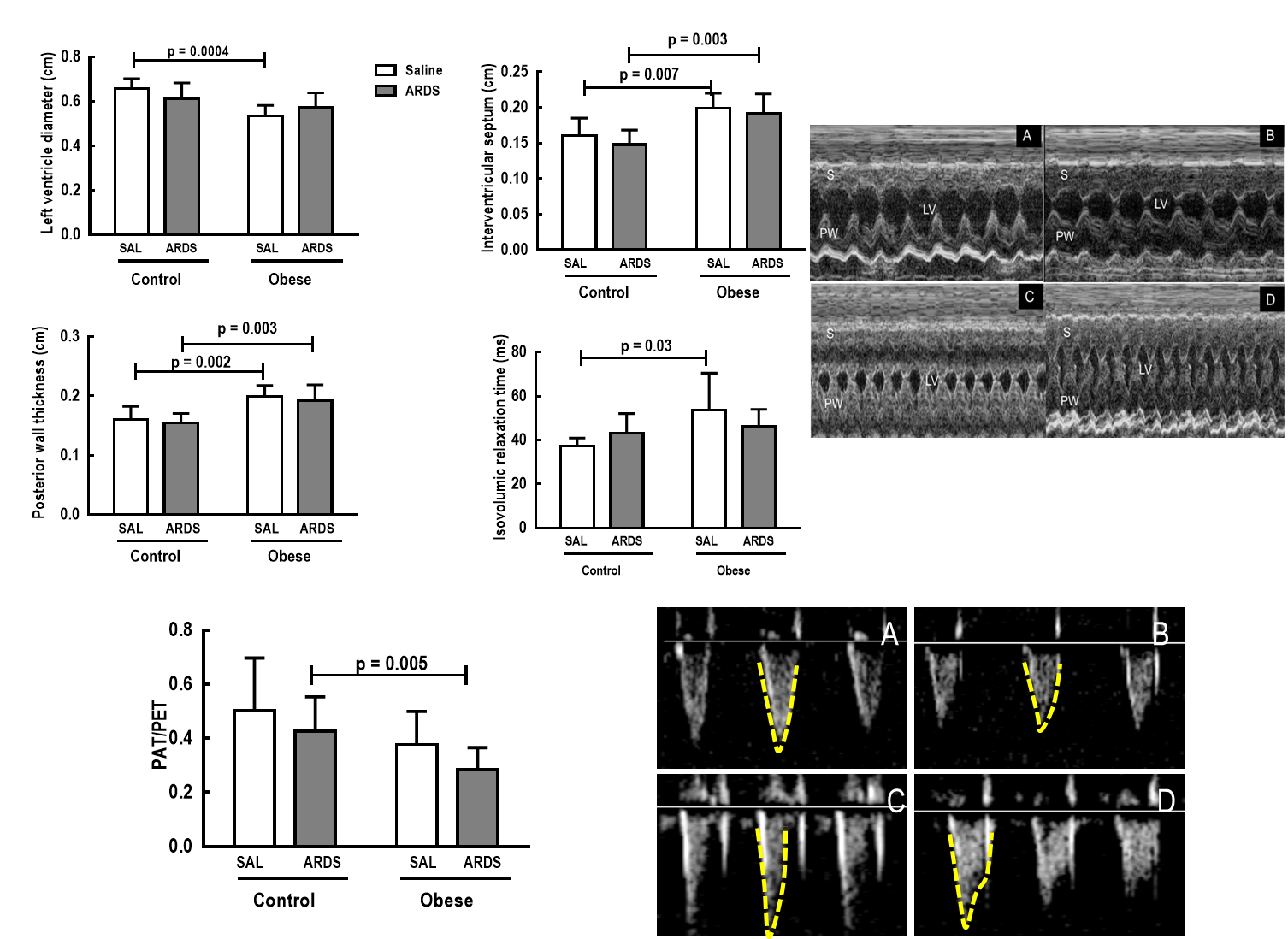


**Supplemental Figure S2:** Left panels: Left ventricular internal diameter, interventricular septum thickness, posterior wall thickness, isovolumic relaxation time, and pulmonary acceleration time/pulmonary ejection time ratio in Control and Obese animals treated with saline (SAL) or endotoxin (ARDS) (n=9 each). Values are means; vertical bars represent the standard deviation. Right panels: M-mode image, short-axis view of left ventricle (note presence of left ventricular concentric hypertrophy in Obese animals), and pulmonary artery Doppler. A = Control-SAL; B = Control-ARDS; C = Obese-SAL; D = Obese-ARDS; S = septum; PW = posterior wall; LV = left ventricle.
