# Supplementary material for: Effects of Obesity on Pulmonary Inflammation and Remodeling in Experimental Moderate Acute Lung Injury
Source: Front Immunol. 2019 May 29;10:1215. doi: 10.3389/fimmu.2019.01215 (PMC6593291; doi:10.3389/fimmu.2019.01215)
Supplement: Supplementary file 4 [file Data_Sheet_3.docx]

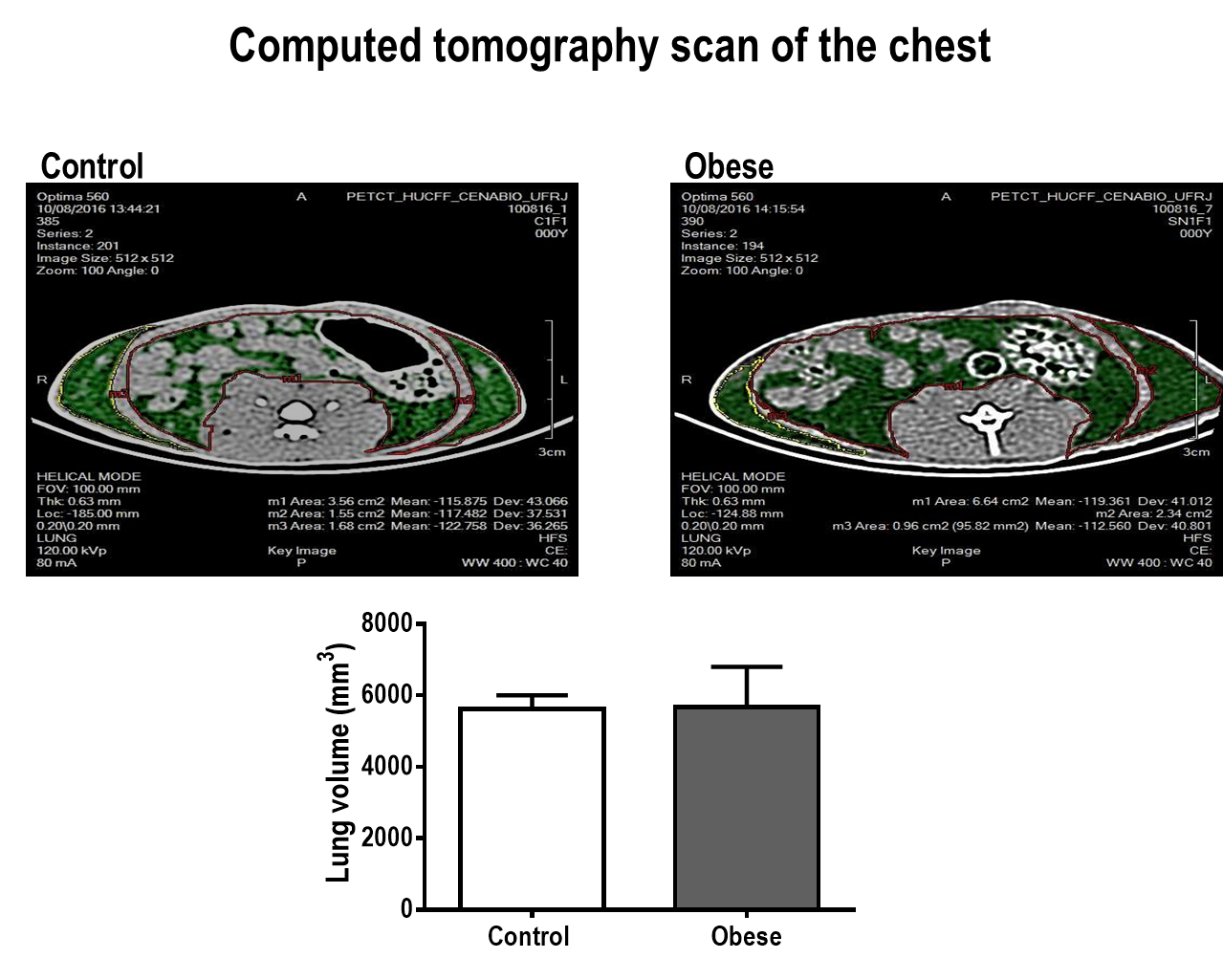


**Supplemental Figure S3.** Computed tomography scan of the chest in Control and Obese animals (upper panels). Lung volume in Control and Obese animals (bottom panel). Values are means + SD of 18 animals/group.
